# Which pronator are you? New perspectives from an unsupervised clustering approach in running

**DOI:** 10.3389/fspor.2025.1682315

**Published:** 2026-01-12

**Authors:** Nicolas Flores, Cédric Yves-Marie Morio

**Affiliations:** Foot Motion Lab, Decathlon SportsLab, Lille, France

**Keywords:** calcaneal eversion, injury risk, motion control, overpronation, running, subtalar, tibial rotation

## Abstract

**Introduction:** Excessive pronation is still considered as a factor partially involved in the running-related injury risk despite inconsistent evidence. The between-runner variability and the way excessive pronation is quantified are potentially involved issues. The purpose of this study is to highlight the different functional pronation movements in runners to be able to propose suitable and up-to-date excessive pronation thresholds.

**Methods:** 234 (overground) and 190 (treadmill) recreational runners ran at a self-selected speed while the lower limb dynamic pronation was measured with skin-mounted markers to calculate seven common pronation-related kinematic variables of the rearfoot and the tibia.

**Results:** These variables were shown to provide different, complementary, information regarding pronation, which influenced the unsupervised hierarchical clustering. Three distinctive functional pronation movements were identified: fast with large excursions (cluster 1), prolonged with high peaks (cluster 2), and overall low (cluster 3). Excessive pronation thresholds were proposed from the typical variables of clusters 1 and 2: −16.9° of maximal rearfoot eversion, 25.8° of rearfoot excursion, −10.0° of maximal tibia internal rotation, 20.2° of tibia internal rotation excursion, −849 °/s of maximal rearfoot eversion velocity, 0.273 s of rearfoot eversion duration, and 1.18 of ratio of excursion of the rearfoot eversion to the tibia internal rotation.

**Discussion:** To get greater evidence of pronation involvement in injury risk, future studies should refer to these results to separate runners with and without excessive pronation for studying the effect of a given intervention on these groups and/or for assessing them in longitudinal follow-up studies.

## Introduction

Pronation has been one of the most important topics of interest in the scientific, clinical, and industrial fields for more than 40 years due to its putative, yet unclear, implications in running-related injuries. While pronation is a natural and universal movement occurring during locomotion (1), excessive pronation has been cited as a potential risk factor in injury risk during running (2–5).

The concept of excessive pronation is based on quantitative thresholds that have been exceeded in a determined amount in the kinematics of skeletal movements. Bone-mounted markers (6) or x-rays (7) are the most effective ways to quantify true movements around the subtalar joint. A more common non-invasive way is obtained from skin-mounted (or shoe) markers. This last allows an indirect pronation quantification around the longitudinal axis of the foot (1), in terms of three-dimensional rearfoot kinematics relative to the tibia, and inversely (8). Usual pronation-related kinematics variables are related to rearfoot eversion (maximal, excursion, velocity, and duration), tibia internal rotation (maximal and excursion), and the combination of both (ratio). These variables aim to consider the complex combination of coupling movements of different segments during pronation (6, 9–11). To the authors' knowledge, available dynamic-based excessive pronation thresholds only exist for maximal rearfoot eversion [−13° in (12) or −18° in (13)] and rearfoot excursion [19° in (12)]. Neither the tibia kinematics nor the temporality dimension is considered. Yet, the tibia kinematics is shown to be relevant for distinguishing runners with or without anterior knee pain (14), and the rearfoot duration is the pronation-related variable involved with the greatest evidence in medial tibial stress syndrome (15). Not considering these pronation-related variables in the setting of excessive pronation thresholds could be an issue in the strategy of understanding mechanisms and injury prevention during running.

The pronation coupling movements between lower-limb segments are high but not perfect (9). A given amplitude of movement of the rearfoot is not fully proportional to the movement of the tibia. Furthermore, this coupling varies between individuals by twice as much (6, 9, 10), and the pronation coupling patterns are quite runner-specific (6). In addition to the potential incomplete set of variables for setting excessive pronation thresholds, these thresholds should thus be individually fine-tuned (16), which is very challenging. A more feasible compromise could be to use a functional grouping approach on pronation movements (3, 17). Such an approach would enable obtaining groups with different pronation characteristics, with each group gathering individuals who share similar pronation-related kinematic variables. By considering more the variety of between-individual pronation movements, this approach may bring new insights for injury-prevention strategies, by targeting one specific pronation group or sub group of excessive pronators above predefined risk-threshold.

The main purpose of this study is to highlight functional groups of pronation during running. A secondary purpose is to set excessive pronation thresholds based on the clustering results. Given the quite high number of kinematic variables available to characterise pronation of the rearfoot and the tibia, it was hypothesised that the clustering approach would provide several clusters differing in the variable types (angle- vs. temporal-dependent) and/or the lower limb segments (rearfoot vs. tibia). Thresholds for pronation-related kinematic variables would be set from the subsets of individuals with the highest values for the main variables characterising each pronation functional group.

## Methods

### Participants

A dataset of 424 runners was considered, including overground (203 males, 31 females; 30.4 ± 8.3 yrs, 176.3 ± 6.4 cm, 71.2 ± 10.2 kg, 11.2 ± 1.4 km/h) and treadmill (142 males, 48 females; 33.5 ± 9.3 yrs, 176.2 ± 7.6 cm, 71.9 ± 10.9 kg, 10.1 ± 1.0 km/h), to reflect the different facilities of laboratories working on this pronation topic. The present dataset was retrospectively extracted from previous unpublished studies from our research group. The study inclusion criteria were that they have to use the same methodology and standard footwear characteristics, described as follows. The participants were free from injury in the six months preceding the participation in the study. All participants gave their written informed consent to the procedures performed during the study in accordance with the institutional ethical rules of the authors' affiliation. All experiments were performed in accordance with relevant guidelines and regulations and the Declaration of Helsinki.

### Markerset

**Figure 1:**
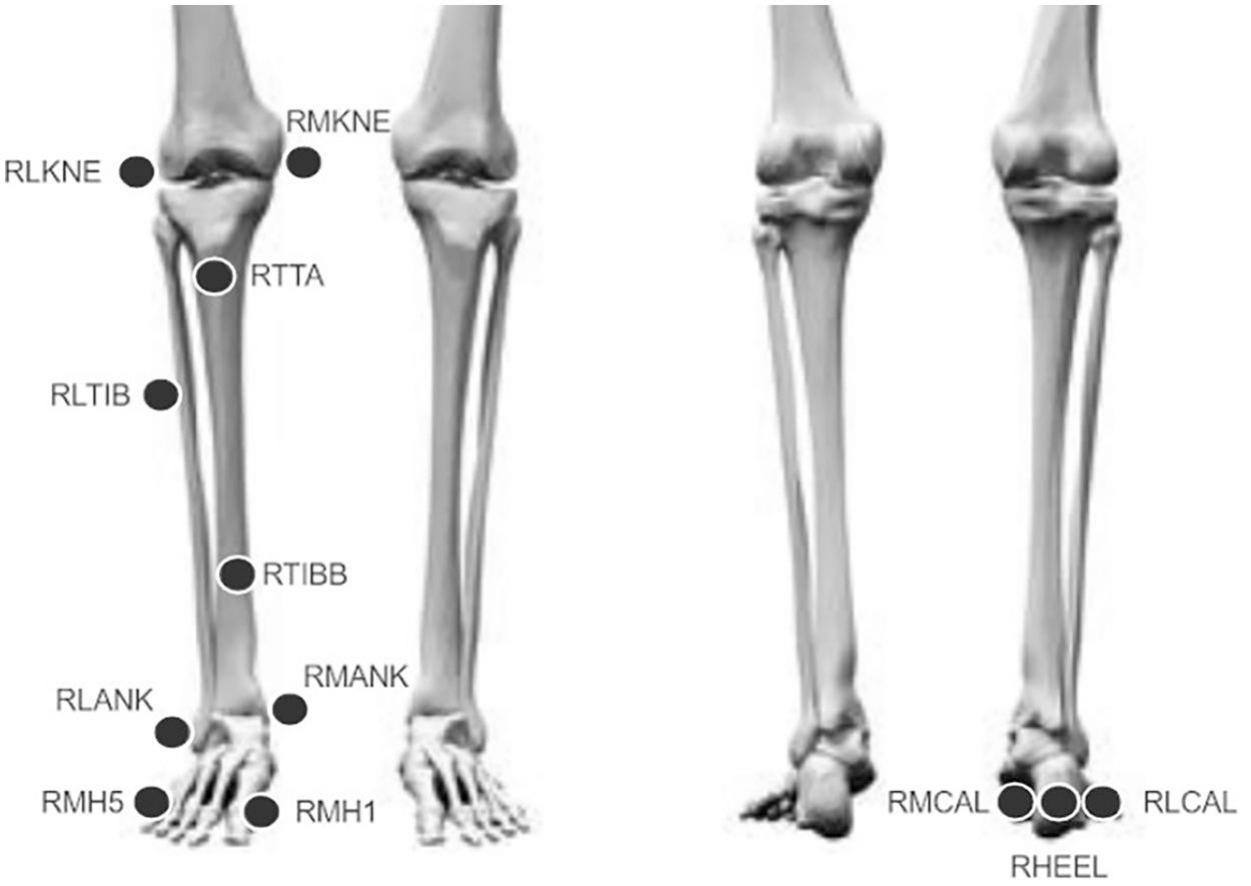
Setting of the skin-mounted markers.

Participants were equipped with twelve skin-mounted retroreflective markers on one lower limb either left or right depending on the sub part of the dataset (Figure 1). Five two-centimetre holes (18) were cut into the footwear upper to allow the marker placement on the foot skin (three on the calcaneus and two on the metatarsal heads). The footwear was neutral, without any specific stability or motion control features, and was provided to the participants.

### Overground experiment

This part only concerns the 234 runners. A 10 min warm-up on a hard concrete floor was performed at a self-selected comfortable running speed. The average warm-up speed was measured with timing gates. After a brief rest to equip the participants with markers, a static standing trial was recorded, and participants ran continuously for 5 min on the runway with the provided experimental neutral footwear, at the target speed (±5%) measured during the warm-up. The run was then prolonged until five valid trials were recorded. The trajectory of the markers was recorded at 200 Hz (Oqus series, Qualisys, Sweden) and the ground reaction forces at 2,000 Hz (9287 CA, Kistler, Switzerland).

### Treadmill experiment

This part only concerns the 190 runners. The treadmill (T900D, Domyos, France) has been stiffened with steel bars to obtain a running surface closer to an overground stiffness. A 10 min warm-up on the treadmill was performed at a self-selected comfortable running speed. The researcher manipulated the treadmill speed based on the participants' feedback until they reached their declared comfortable speed. After a brief rest to equip the participants with markers, a static standing trial was recorded, and participants ran continuously at the target speed for 5 min on the treadmill with the provided experimental neutral footwear. The run was prolonged for one minute, the last thirty seconds allowed the measurements of the marker trajectories at 200 Hz (Oqus series, Qualisys, Sweden) corresponding to a minimum of ten strides.

### Data analysis

The lower limb model consisted of two segments (v2024.02.2, Visual3D, HAS-Motion, Canada). The rearfoot segment was defined proximally by the mid-distance between the ankle malleolus markers, and distally by the first (medial) and fifth (lateral) metatarsal head markers, and the three calcaneus markers were used for the tracking of the rearfoot segment during running trials. The tibia segment was defined proximally by the medial and lateral knee condyle markers, and distally by the mid-distance between the ankle malleolus markers, and the three tibia markers were used for the tracking of the tibia segment during running trials. Marker trajectories were low-pass filtered with a double-pass zero-lag 2nd order Butterworth-type filter at a 30 Hz cut-off frequency. For the overground experiment, the ground reaction forces were filtered at a 50 Hz cut-off frequency with a 5 double-pass zero-lag 2nd order critically damped filter. The rearfoot eversion was computed as the frontal rotation of the rearfoot segment in the tibia segment coordinate system. The tibia internal rotation was computed as the transverse rotation of the tibia segment in the rearfoot segment coordinate system. The static standing trial was used as the zero reference position for the angle calculations. The XYZ Cardan sequence was used for both Euler angle calculations, with X the mediolateral axis, Y the anteroposterior axis, and Z the segment longitudinal axis.

The foot contact on the ground was identified with two distinct methods depending on the available measurement devices into the overground and treadmill experiments in the current study, like it could be the case in different lab facilities. For the overground dataset, the ground contact was identified in a gold standard way from the force plates signals, when the ground reaction force in the vertical component was greater than 5 N. For the treadmill dataset, kinematics signals were used as the current treadmill was not instrumented with force sensors. The foot touchdown was identified as the maximum anterior distance reached by the posterior calcaneus marker. The toe-off was identified as the virtual distal foot marker, equidistant on a line relying on the markers of the first and the fifth metatarsal heads, exceeding 0.07 m vertically above the treadmill belt. This height has been identified on average at the instant of the toe-off from the overground dataset, to ensure some consistency with the gold standard method. The instants of the first and last ground contacts were used to cut the kinematics signals.

**Figure 2:**
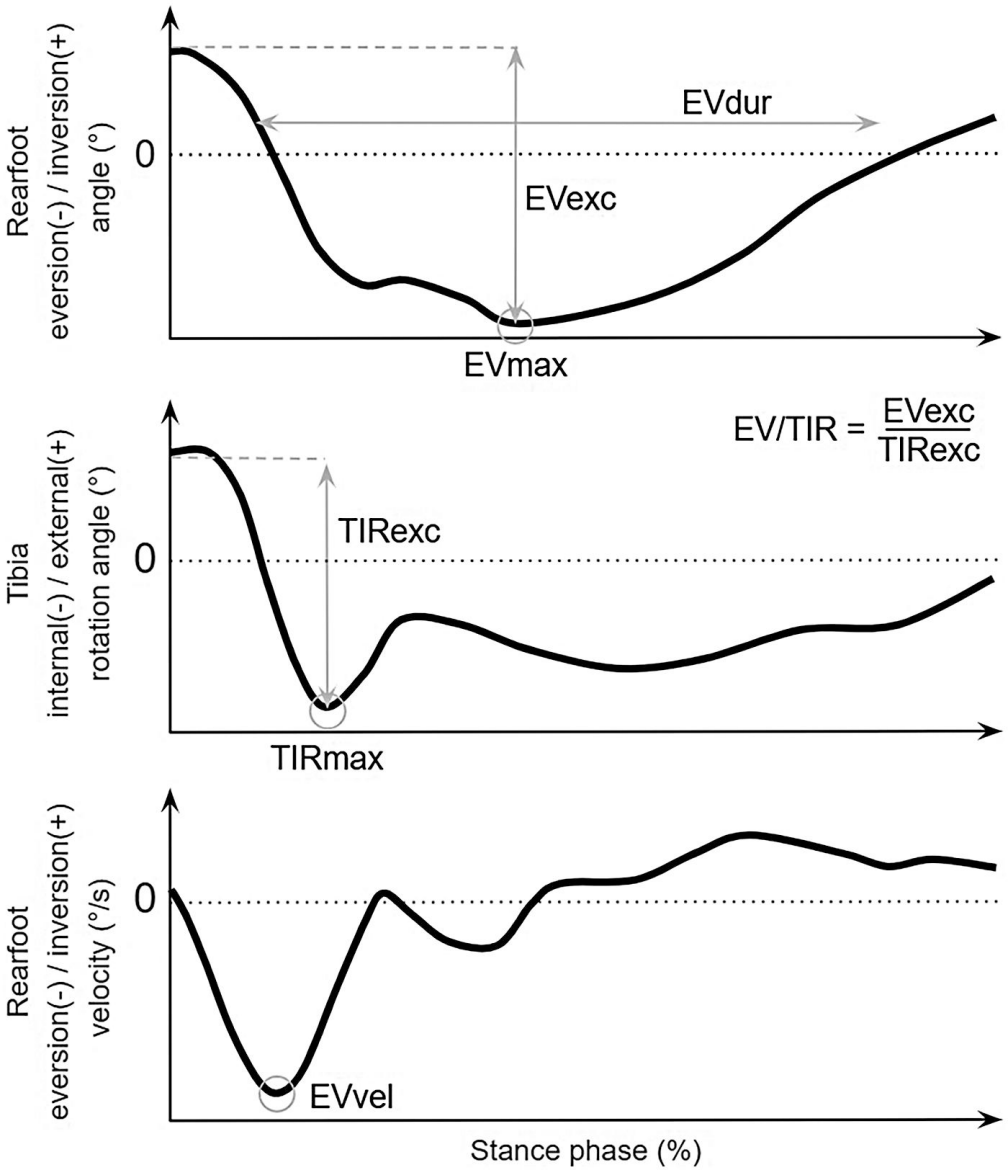
Pronation-related kinematic variables computed from typical rearfoot eversion/inversion angle (top), tibia internal/external rotation angle (middle), and rearfoot eversion/inversion velocity (bottom) curves.

Seven pronation-related kinematic variables (Figure 2) were finally computed on a homemade Matlab script (R2024b, Mathworks, Inc., USA). Maximal rearfoot eversion (EVmax) and maximal tibia internal rotation (TIRmax) were identified as the minimum angle value of both angles during the stance phase. Excursion of rearfoot eversion (EVexc) and tibia internal rotation (TIRexc) were computed as EVmax and TIRmax minus the corresponding value at the instant of the first ground contact. The ratio of these excursions was computed (EV/TIR). The duration of rearfoot eversion (EVdur) was quantified as the duration during which the rearfoot angle was negative during the stance phase. The rearfoot eversion angle was derived to obtain the rearfoot eversion velocity, and the maximal rearfoot eversion velocity (EVvel) was identified as the minimum value during the first half of the stance phase. Each variable was finally averaged among all the trials recorded for each participant.

### Statistical analysis

A dataframe of 7 variables for the 424 participants was used for the subsequent analysis in RStudio (v2024.09.1, USA) with the “*FactoMineR*” package (19). A principal component analysis (“*PCA*” function) was first performed on the data centered and scaled to unit variance. An unsupervised hierarchical clustering on principal components (“*HCPC*” function) was then performed with Euclidean distances and the Ward agglomeration method that is suitable for multidimensional variance (i.e., inertia). The Ward method aggregates two clusters such that the growth of within-inertia is minimum, and the inertia inter clusters is maximized, at each step of the algorithm. The criterion automatically used by the function for defining the partition (i.e., cluster number) is the higher relative loss of inertia. Explained variance in the principal components (PC) and the percentage of contribution of the variables in each principal component were obtained from the “*PCA*” function. The number of clusters, the cluster of each participant, and the main variables characterising each cluster were obtained from the “*HCPC*” function. The function basically runs an independent *t*-test on each variable between the mean of a given cluster and the total dataset mean. Significant differences (*p* < 0.05) indicated that the corresponding variables mainly characterised the given clusters, by being either greater or lower than the total dataset mean.

Descriptive statistics in terms of mean, standard deviation, first and third quartiles were calculated for the main variables characterising each cluster. Depending on the sign of the variable, the first (i.e., EVmax, TIRmax, TIRatEVmax, EVvel) or the third (i.e., EVexc, TIRexc, EVdur, EV/TIR) quartile of this variable was used for setting excessive pronation thresholds. For example, if EVexc of a given cluster is significantly greater than the total dataset mean, then the value of the third quartile of EVexc of this cluster is kept for setting the excessive pronation threshold of EVexc. In other words, the excessive pronation threshold of EVexc is based on the 25th percentile of the runners, who are already characterised with high EVexc in a given cluster by the clustering algorithm. Choosing the quartile criterion enables being less sensitive to the normal or skewed distribution of the data for setting excessive pronation thresholds in each variable (Supplementary Material A).

## Results

**Figure 3:**
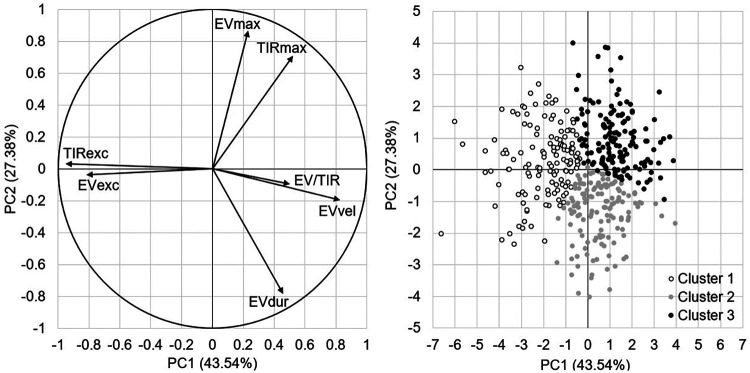
(Left) Principal component analysis graph of variables on the first two PCs. Arrows oriented towards the same or the opposite directions are respectively positively or negatively correlated, while the correlation is low when variables are oriented in different directions. Note that EVmax, TIRmax, and EVvel have a negative sign. (Right) Unsupervised hierarchical clustering coordinate graph of individuals on the first two PCs.

All the information regarding the variance in the data from the seven PCs as well as the contribution of each variable to each PC is presented in Supplementary Material B. For more clarity, we presented hereafter the main variables contributing to the three first PCs, which explained 87.4% of the variance in the data. TIRexc (30%), EVVel (22%), and EVexc (22%) mainly contributed in the first PC, EVmax (39%), EVdur (32%), and TIRmax (26%) in the second PC, and EV/TIR (61%) and EVexc (19%) in the third PC (Figure 3). Three clusters emerged from the unsupervised hierarchical clustering (Figure 3).

The first cluster of 134 individuals is characterised by significantly more TIRexc (+4.7°; *p* = 6.0*e^−54^), EVvel (−255°/s; *p* = 3.3*e^−47^), EVexc (+4.7°; *p* = 5.6*e^−40^), and TIRmax (−1.7°; *p* = 1.2*e^−11^), and less EVdur (−0.038 s; *p* = 7.2*e^−20^) and EV/TIR (−0.16; *p* = 4.0*e^−12^), compared to the total dataset mean (Table 1). Pronation in cluster 1 is functionally fast with large excursions in the rearfoot eversion and the tibia internal rotation, and high involvement of the tibia internal rotation relative to the rearfoot eversion.

**Table 1:** Means (standard deviations) of the seven pronation-related kinematic variables of the total dataset, and the three functional pronation clusters.

Variable	Total dataset *n* = 424	Cluster 1 *n* = 134	Cluster 2 *n* = 138	Cluster 3 *n* = 152
EVexc (°)	18.6 (5.0)	23.3 (4.5)* Q3 **=** **25.8**	17.5 (3.3)*	15.4 (3.2)*
TIRexc (°)	13.0 (4.2)	17.7 (3.3)* Q3 **=** **20.2**	11.7 (2.4)*	10.1 (2.5)*
EV/TIR	1.48 (0.30)	1.33 (0.20)* Q1 **=** **1.18**	1.54 (0.32)*	1.57 (0.32)*
EVmax (°)	−12.3 (3.9)	−12.5 (3.5)	−15.2 (3.1)* Q1 **=** **−16.9**	−9.4 (2.5)*
TIRmax (°)	−6.6 (3.2)	−8.1 (2.8)*	−8.2 (2.2)* Q1 **=** **−10.0**	−3.9 (2.3)*
EVvel (°/s)	−484 (247)	−739 (264)* Q1 **=** **−849**	−371 (94)*	−362 (133)*
EVdur (s)	0.202 (0.052)	0.166 (0.037)*	0.250 (0.041)* Q3 **=** **0.273**	0.189 (0.039)*

* Indicates a significant difference of the variable between the given cluster and the total dataset mean. Q1 and Q3 in bold indicate the first and the third quartile values to define excessive pronation thresholds.

The second cluster of 138 individuals is characterised by significantly more EVdur (+0.049 s; *p* = 5.2*e^−40^), EVmax (−2.9°; *p* = 3.0*e^−27^), TIRmax (−1.6°; *p* = 4.0*e^−13^), and EV/TIR (+0.06; *p* = 4.0*e^−12^), and less EVvel (+112°/s; *p* = 6.4*e^−11^), TIRexc (−1.4°; *p* = 5.5*e^−6^), and EVexc (−1.1°; *p* = 2.3*e^−3^), compared to the total dataset mean (Table 1). In cluster 2, pronation is functionally prolonged with high angle peaks in the rearfoot eversion and the tibia internal rotation.

The third cluster of 152 individuals is characterised by significantly less TIRmax (+2.7°; *p* = 4.5*e^−40^), EVmax (+2.9°; *p* = 1.4*e^−30^), TIRexc (−2.9°; *p* = 4.9*e^−26^), EVexc (−3.2°; *p* = 1.8*e^−17^), EVvel (+122°/s; *p* = 2.8*e^−14^), EVdur (−0.013 s; *p* = 1.6*e^−4^), and more EV/TIR (+0.09; *p* = 1.2*e^−5^), compared to the total dataset mean (Table 1). In cluster 3, pronation is functionally overall low.

The excessive pronation thresholds were set from clusters 1 and 2 (Table 1). From cluster 1, the thresholds are 25.8° for EVexc, 20.2° for TIRexc, −849°/s for EVvel, and 1.18 for EV/TIR. From cluster 2, the thresholds are −16.9° for EVmax, −10.0° for TIRmax, and 0.273 s for EVdur.

## Discussion

This study aimed to highlight the different functional pronation movements in runners in order to propose suitable and up-to-date excessive pronation thresholds. Pronation in runners has been classified into three main functional movements: fast with large angle excursions, prolonged with high angle peaks, and overall low. From the classification results, excessive pronation thresholds have been proposed for each pronation-related kinematics variable, which challenged and enriched the previously existing thresholds.

Principal components analysis shows that the pronation-related kinematic variables provide different information about the complex dynamics of pronation. Peak variables (EVmax and TIRmax) share quite similar information, which is different from excursion and velocity variables (EVexc, TIRexc, and EVvel), while duration (EVdur) or excursion ratio (EV/TIR) variables also provide different information. This result is in line with a previous study showing high correlations within a set of pronation variables using the same basic definition, but poor correlations between sets of variables using different definitions (20). This highlights the need to quantify pronation from multiple complementary variables to consider different aspects of the complex combination of coupling movements of the lower limb segments during running. Whether coupling movements between the rearfoot and tibia segments are not perfect (9), they seem to be high enough (11) as the current results suggest that information is quite similar between both segments. This means that, within the same variable type (i.e., peak or excursion), measuring the rearfoot eversion or the tibia internal rotation is similar, thus only measuring one of these segments would be enough. However, it remains important to measure both the rearfoot and tibia kinematics because the ratio of excursion of these segments (EV/TIR) provides different information from other pronation-related variables.

The unsupervised classification is directly influenced by the different information provided by the pronation-related kinematic variables. Three clusters of runners with distinct functional pronation movements are obtained: fast with large excursions (cluster 1), prolonged with high peaks (cluster 2), and overall low (cluster 3). The different pronation characteristics of these clusters suggest that the running-related injury risks could be different between clusters.

The characteristic variables of cluster 1 are large EVexc, large TIRexc, fast EVvel, and low EV/TIR. Large EVexc and fast EVvel have been shown to be correlated with high Achilles tendon force loading rate and tibiofemoral compressive force impulse (21). Large and fast rearfoot movements may load the Achilles tendon due to a whipping action (22), restricting the tendon blood flow (23), likely explaining why these variables are reported to be involved in the injury risk, especially for Achilles tendinopathy (15, 24). The whipping action could also be amplified through a large amount of tibia internal rotation, which has been shown to lower EV/TIR ratio in pronator runners (8) and in runners with anterior knee pain (14). Besides being detrimental for the Achilles tendon, this low EV/TIR is also suggested to have impact at the knee (8). Following these previous results, it is speculated that functional pronation of cluster 1 would expose runners to greater injury risk in the Achilles tendon and knee structures, which should be tested in future studies.

The characteristic variables of cluster 2 are high EVmax, high TIRmax, and prolonged EVdur. High EVmax is supposed to be involved with inconsistent/limited evidence in tibial stress fracture and medial tibia stress syndrome (15), and recently with plantar fasciitis in a prospective study (25). Medial tibia stress syndrome has been proposed to be the result of traction of soleus, posterior tibial, and flexor digitorum longus muscles on the tibia periosteum (26). The potential traction of these muscles might be due to the frontal misalignment of the foot-leg segments, especially in presence of high rearfoot eversion. This misalignment can increase the external lever arm of the ground reaction force resultant relative to the ankle joint center in the frontal plane (27), thus inducing more activity or force of these muscles to control and perform the task. It would be relevant to test whether the pronation variables of cluster 2 (i.e., EVmax, TIRmax, and EVdur) are correlated to the muscle force estimations of the soleus, posterior tibial, and flexor digitorum longus from musculoskeletal modelling. This would bring more evidence of these pronation variables in the aetiology of medial tibia stress syndrome. Currently, only prolonged EVdur is suggested to be involved with the highest evidence in medial tibial stress syndrome (15). A prolonged EVdur may be detrimental for the tibia structures but might unload other leg structures as prolonged EVdur was correlated to low Achilles tendon force peak and loading rate (21). Future studies could test the speculative assumption that functional pronation of cluster 2 might expose runners to greater injury risk in the tibia and foot structures, and might be protective for the Achilles tendon structure.

In the current study, the type of pronation-related kinematic variables is the driver of the classification rather than the intensity of pronation. This questions the relevance of the very popular way to segment pronation in terms of intensity, basically as underpronation, normal/intermediate, or excessive/overpronation. Even if pronation is much more important in clusters 1 and 2 than in cluster 3, it is not possible to assume that pronation is more excessive in cluster 1 than in cluster 2 (or inversely). Pronation can be excessive both in cluster 1 or 2 depending on the variables of interest. For this reason, excessive pronation thresholds have been set on each of the seven pronation-related kinematic variables based on the specific variables characterising the clusters 1 and 2.

The current available thresholds in the literature for excessive pronation have been only set on EVexc and EVmax (12, 13). The thresholds set by Clarke et al. on EVexc (19°) and EVmax (−13°) correspond to the values of the total dataset means of the current study (18.6° and −12.3°, respectively). These thresholds have been set several decades ago when the common way to measure pronation was different. Back view videos tracked the trajectories of two markers on the calcaneus and two markers on the lower leg. These markers were used to define one line for the rearfoot and one line for the leg, with the rearfoot angle being the difference of the two line angles in the frontal plane of the running direction (12). This two-dimensional measurement combined with the fact that calcaneus markers were almost exclusively located on the shoe heel counter (28–30) can explain why the previous thresholds could be underestimated. For these reasons, it is suggested that these previous thresholds should not be considered anymore. From calcaneus skin-mounted markers, with modern motion capture devices and computation of pronation variables in three-dimension, the new proposed thresholds are set to 25.8° for EVexc based on runners in cluster 1 and −16.9° for EVmax based on runners in cluster 2. The threshold of EVmax is near to the slightly more conservative previous threshold of −18° in McClay and Manal (13). Thanks to the high sample size (*n* = 424), on two running surfaces typically used in different lab facilities (overground and treadmill), in a high range of running speeds (from 7.5 to 15 km/h), it is assumed that the current −16.9° EVmax threshold is more representative of the population of runners. To complete the set of pronation-related kinematics variables, the other proposed thresholds are 20.2° for TIRexc, −849°/s for EVvel, and 1.18 for EV/TIR based on runners in cluster 1, and −10.0° for TIRmax and 0.273 s for EVdur based on runners in cluster 2. These other thresholds have never been set in any previous study, but some can be compared to provided values in the literature in specific runner samples. For example, the value of 1.18 of EV/TIR is a bit more conservative but near to the value of 1.23 reported in pronator runners (8), while the value of 0.273 s for EVdur expressed relatively to the stance time is 81%, which is quite close to the values of 85%–86% measured in runners with medial tibia stress syndrome and Achilles tendinopathy (31). Taken together, the current proposed thresholds are more relevant and should be considered in the future studies for quantifying an excessive pronation in runners.

**Figure 4:**
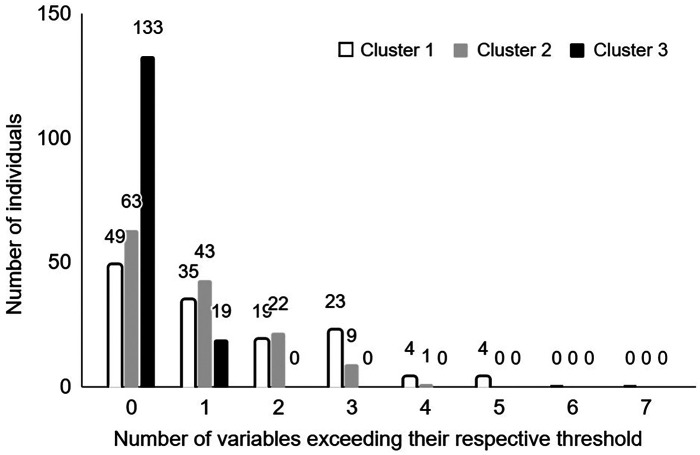
Number of individuals in clusters 1 (white), 2 (grey), and 3 (black) concerned by an excessive pronation as a function of the number of pronation-related kinematic variables exceeding their respective excessive pronation threshold.

**Figure 5:**
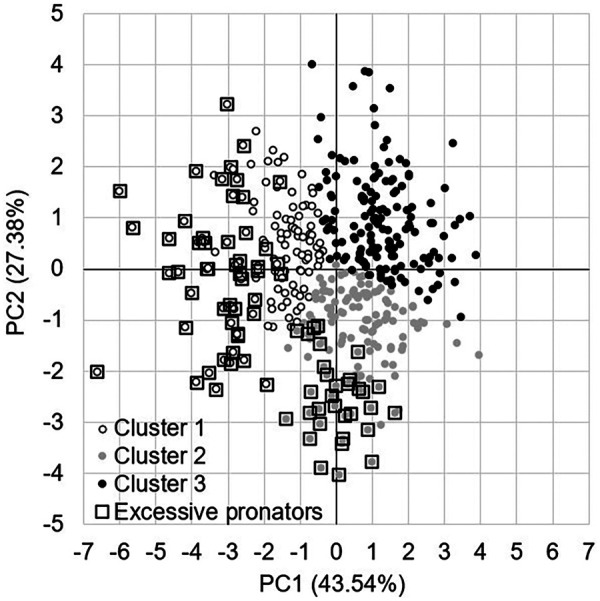
Highlighting of the runners characterised with excessive pronation by considering at least two pronation-related kinematic variables exceeding their respective threshold.

While excessive pronation thresholds are set for all the pronation-related kinematic variables, the remaining question is: when is a runner an excessive pronator? The most direct rationale is to consider an excessive pronator when all the variables exceed the proposed thresholds. Given the results of the current study, this rationale is not possible as the principal component analysis shows that the pronation-related kinematic variables provide different information. Some variables are not correlated or very poorly with each other. This means that no runner can exceed these thresholds simultaneously on all the variables. In the current dataset, at the maximum, five out of seven variables exceed their respective threshold, and for only four out of 424 runners (Figure 4). Also, considering four out of seven variables exceeding their respective thresholds seems to be too much conservative because it would only concern five out of 424 runners. It is recommended to consider at least two variables that simultaneously exceed their respective threshold to consider an individual runner as an excessive pronator. Setting two variables, 19% of 424 runners from the current study would be considered as excessive pronators, including 37% of runners of cluster 1, 23% of runners of cluster 2, and 0% or runners of cluster 3 (Figures 4 and 5).

Given that the cluster 1 and 2 have different pronation functions (fast with large excursions vs. prolonged with high peaks), one more uncertainty still remains following the setting of excessive pronation thresholds in relation with the associated injury risk. Taking as an example two typical runners of cluster 1, with the first runner exceeds thresholds on two typical variables to his cluster 1 only (e.g., EVvel, EV/TIR), while the second runner exceeds thresholds on one typical (e.g., EVvel) and one atypical (e.g., EVmax) variables to his cluster 1. Would the pronation-related running injury risk (in terms of occurrence, location, type, and severity) be different between both runners? This question cannot be answered yet, but it could be addressed in line with emergent paradigms such as the preferred/habitual movement path (16, 17) and/or with the presence of symptoms in runners (32, 33). It is hoped that the current study results and proposals would enable to tackle such questions in future studies, for examining the role of pronation among the multifactorial aetiology of running-related injury.

This study has some limitations and important methodological points to consider. Because foot markers were skin-mounted, participants' habitual running shoes were not usable and standard neutral running shoes with cut uppers were provided to the participants. These standard shoes may modulate their lower limb biomechanics at the individual level compared to their habitual shoes, but likely not enough to change the individuals' preferred (pronation) movement path (17). Left/right asymmetry in running technique was not investigated here, but it could have an impact on pronation classification. Therefore, in future studies aiming to analyze the relationship between pronation classification and running-related injuries, we recommend performing analyses either on both legs independently or on one identical side only. Rearfoot and tibia pronation calculations were made relative to the static standing position (34). Therefore, the zero neutral position is not the alignment of the calcaneus and tibia lines (12), but the amount of foot-tibia pronation configuration in static posture, which is specific to each individual. Due to the difficulty of measuring the subtalar joint with skin-mounted markers during shod running (1), the measured variables were pronation-related and used as surrogates of the *a priori* true foot dynamic pronation. While this study uses scalar values of common pronation-related kinematic variables, it should be acknowledged that pronation is more complex and takes multiple values over time including some time shift between the rearfoot and tibia kinematics (14). The current proposed excessive pronation thresholds can be used as a reference for comparison in future studies as long as similar data analyses are performed, especially for the cut-off frequency of filters and the way of identifying touchdown and toe-off events while running overground or on the treadmill. Excessive pronation thresholds have been set in absolute values for EVvel (°/s) and EVdur (s), which may be dependent on the runners' speed. However, regression analyses show poor associations of the runners' speed with EVvel (*r* = −0.33) and EVdur (*r* = −0.30), such as only 10.8% and 9.0% of the variation in EVvel and EVdur was explained by the variation of the runners' speed (Supplementary Material C). As a result, the threshold of EVvel was not influenced by the fastest runners and the threshold of EVdur was not influenced by the slowest runners. Using these thresholds, the current data showed that the thresholds were independent enough on the runners' speed, as the difference in running speed between excessive pronators and non-excessive pronators was 0.6 km/h in cluster 1 and 0.2 km/h in cluster 2. The dynamic pronation data were measured in three-dimension with an optoelectronic motion capture system, a widespread device in research lab facilities. However, its cost and the need for technical skills to use it might be less accessible to some clinicians, which makes the use of the present data results not completely universal. To reflect different lab facilities, the current study combined data coming from overground and treadmill running (Supplementary Material D). While it is known that both running surfaces may induce different biomechanical patterns, it has been shown that they should be comparable enough, especially with no significant difference in maximal ankle eversion (35). In line with this, the current classification results were not influenced by the running surface as the overground vs. treadmill proportion of measured runners is constant between-clusters (cluster 1: 55% vs. 45%; cluster 2: 57% vs. 43%; cluster 3: 53% vs. 47%).

## Conclusion

The between-individual variability in pronation during running can be summarized into three main functional pronation movements: fast with large excursions, prolonged with high peaks, and overall low. It is thus relevant to first consider the type of pronation in terms of excursion/velocity or peak/duration, before the need to characterise runners by their intensity of pronation to highlight excessive pronation. From the characteristic variables of the two first functional pronation movements, excessive pronation thresholds have been updated and set for seven common pronation-related kinematic variables. For future studies, researchers are encouraged to refer to these thresholds either in a retrospective or prospective way. In both cases, it will permit separating runners with and without excessive pronation for studying the effect of a given intervention on these groups and/or for assessing them in longitudinal follow-up studies. It is believed the current results will help to get better scientific evidence regarding the potential involvement of dynamic pronation in running-related injury risk.

## Data Availability

The datasets presented in this article are not readily available because data is the property of Decathlon, but could be shared under reasonable request. Requests to access the datasets should be directed to Nicolas Flores, nicolas.flores@decathlon.com
