# Supplementary material for: Which pronator are you? New perspectives from an unsupervised clustering approach in running
Source: Front Sports Act Living. 2026 Jan 12;7:1682315. doi: 10.3389/fspor.2025.1682315 (PMC12832910; doi:10.3389/fspor.2025.1682315)
Supplement: Supplementary file 1 [file Datasheet1.docx]

**Supplementary Material A**


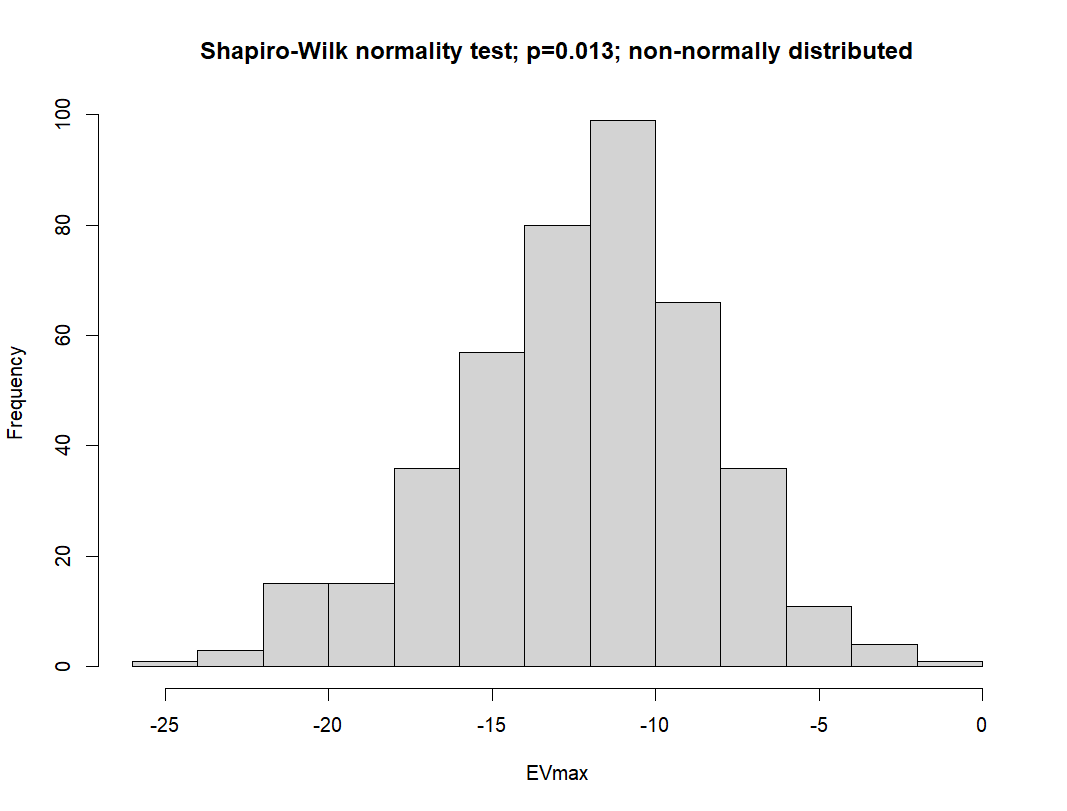

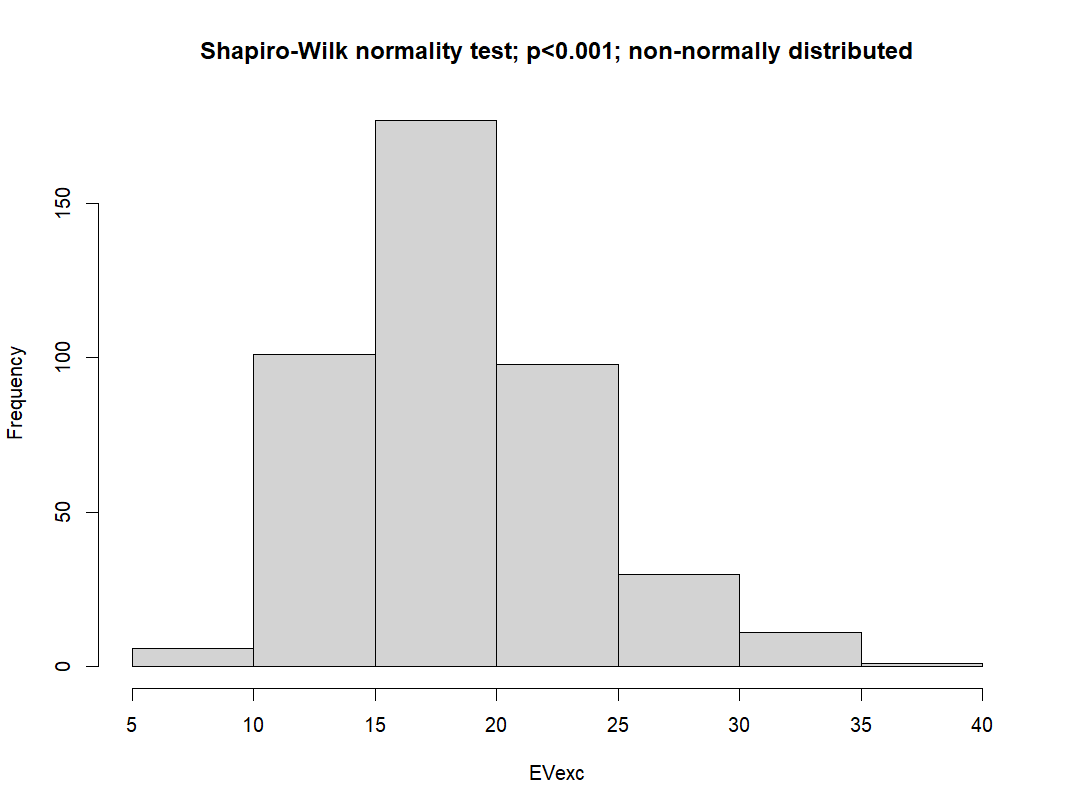


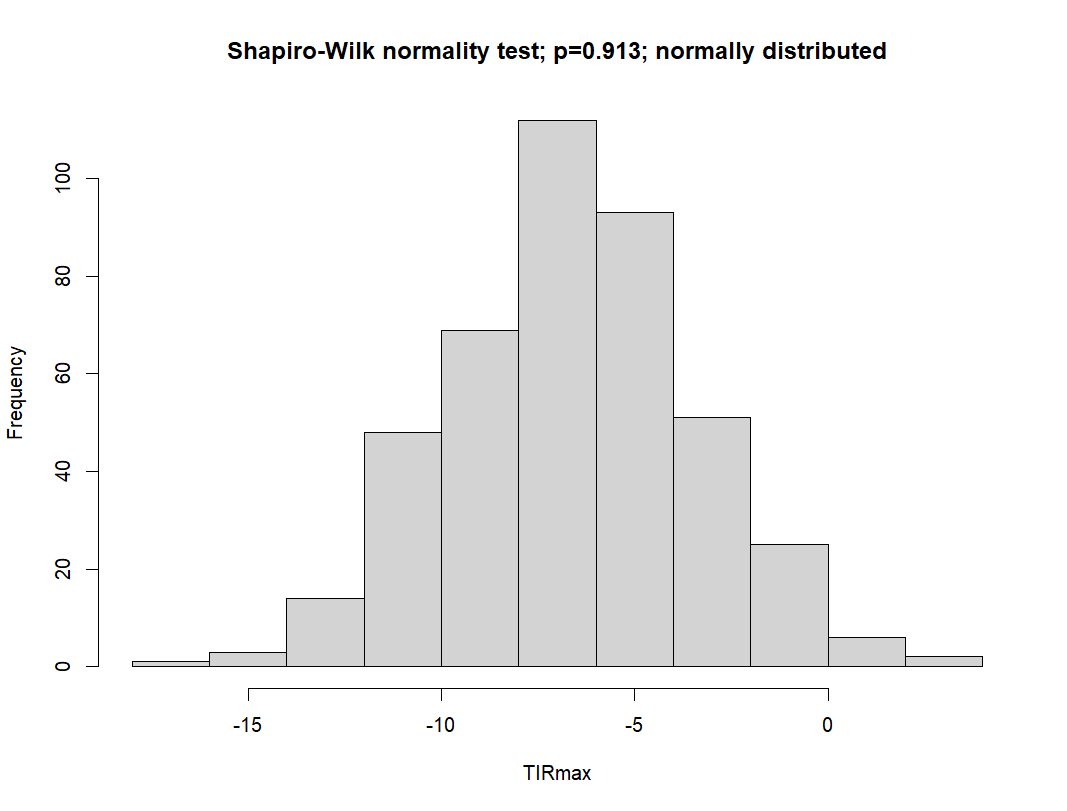

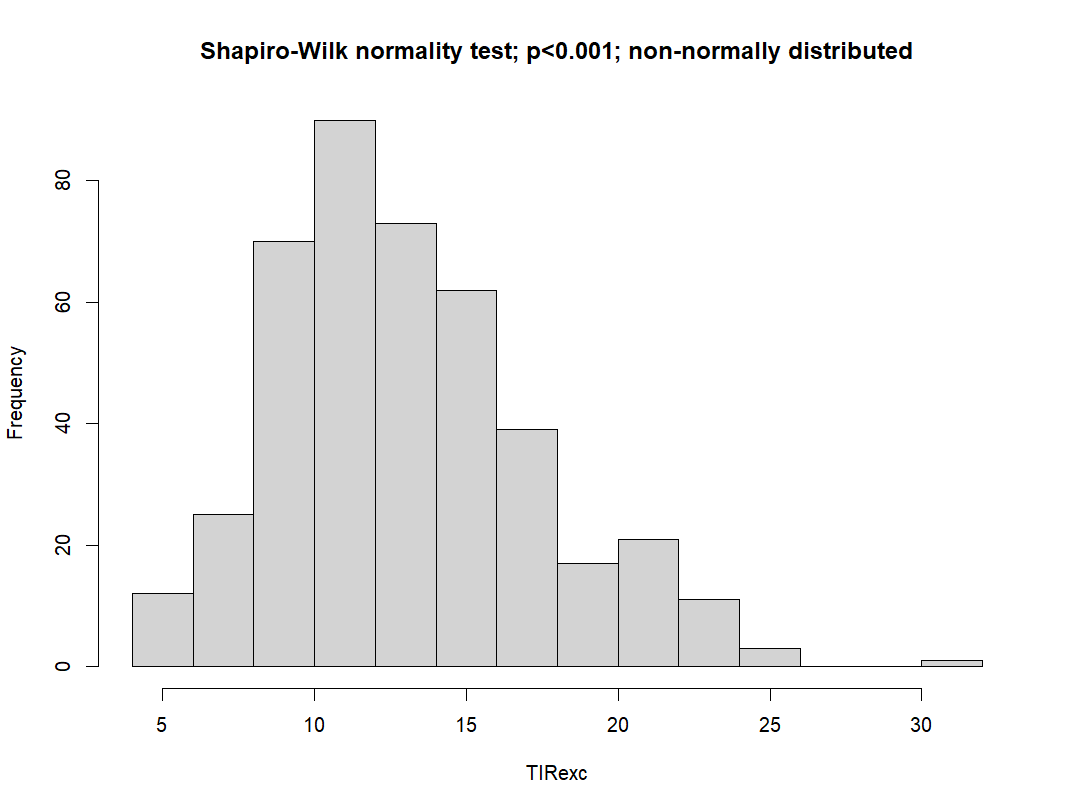

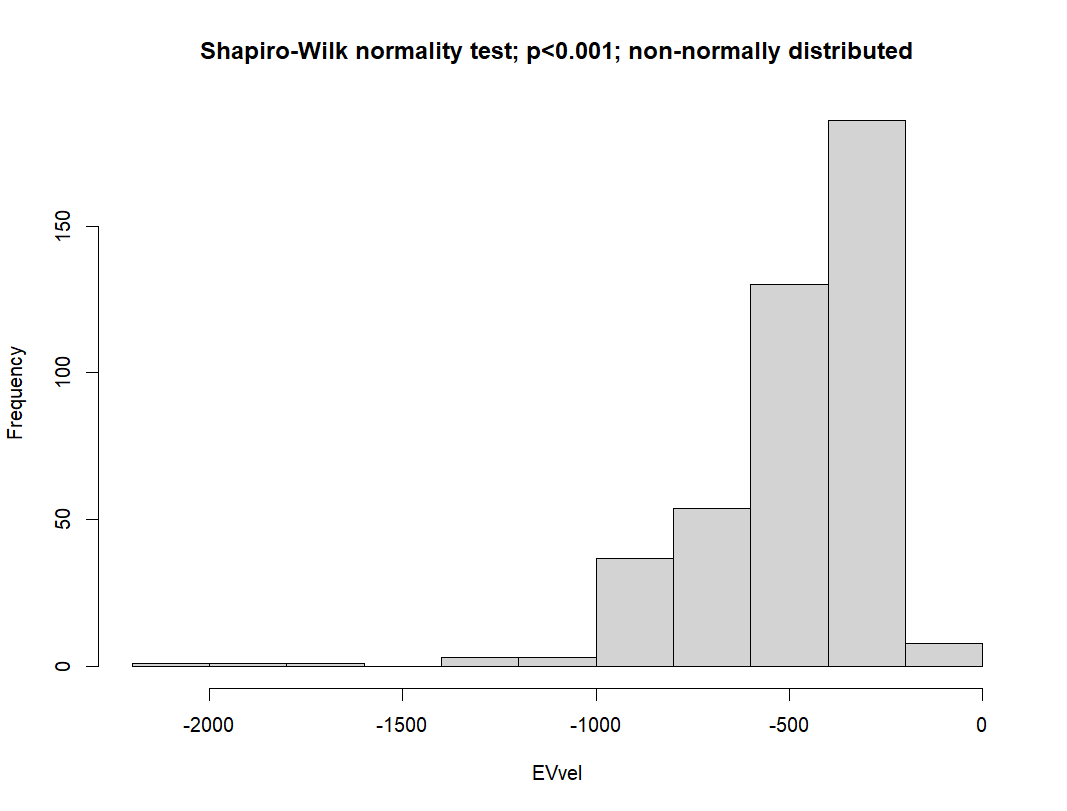

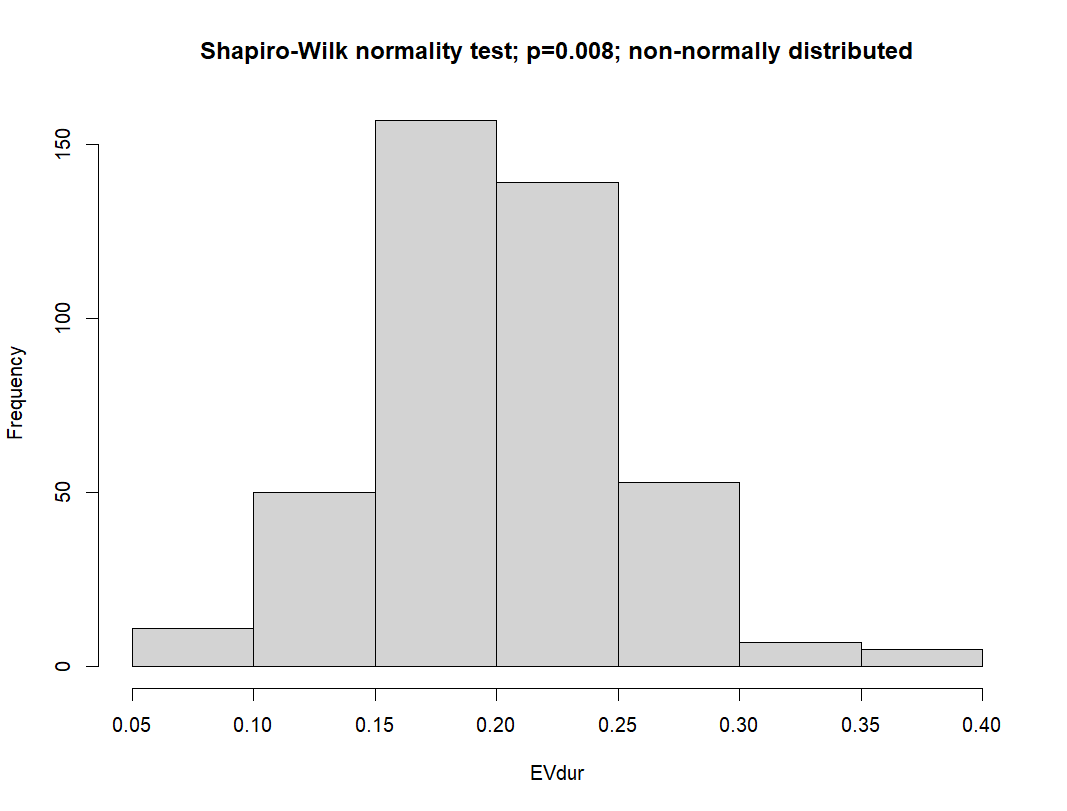


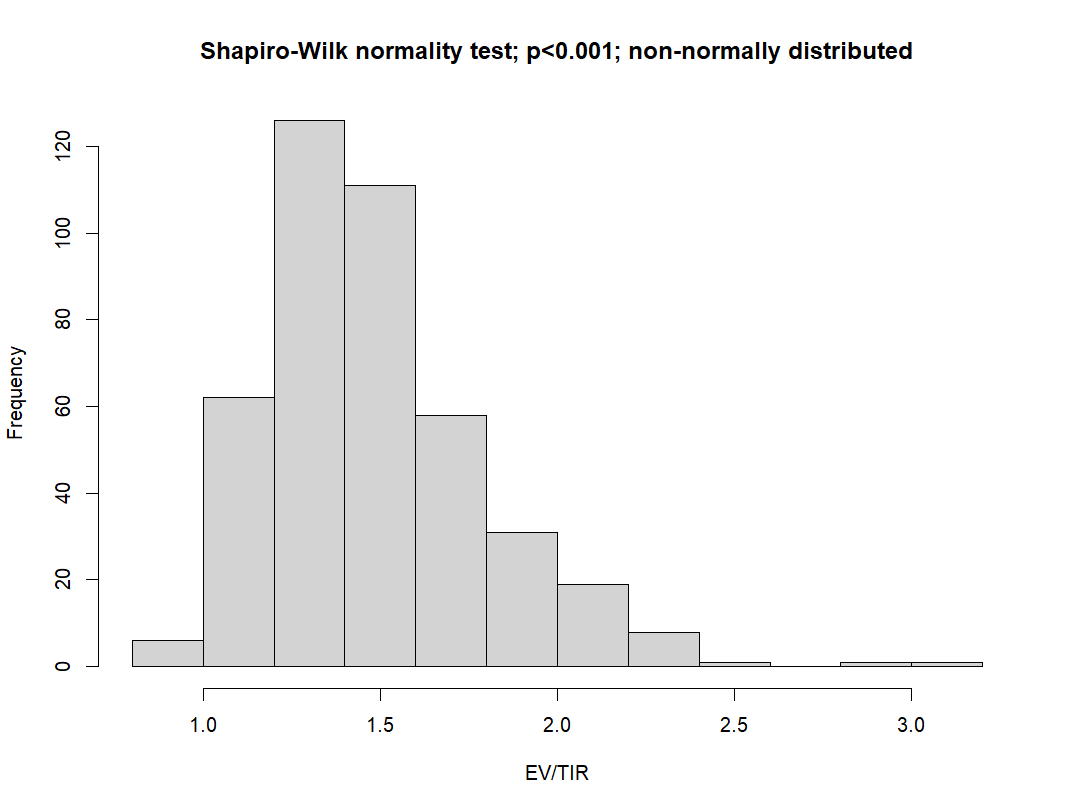


Figure A: Distribution histograms of the participants for each pronation-related kinematic variable with associated results of the Shapiro-Wilk normality test.

**Supplementary Material B**

Table B: Outputs of the principal component analysis in terms of variance explained by each principal component and the contribution of each pronation-related kinematic variable to each principal component.

|  | **PC1** | **PC2** | **PC3** | **PC4** | **PC5** | **PC6** | **PC7** |
| --- | --- | --- | --- | --- | --- | --- | --- |
| **Variance explained (%)** | 43.54 | 27.38 | 16.50 | 6.39 | 3.46 | 2.45 | 0.28 |
| **EVexc contribution (%)** | 21.90 | 0.06 | 18.93 | 23.04 | 0.02 | 1.40 | 34.62 |
| **TIRexc contribution (%)** | 29.74 | 0.06 | 0.48 | 17.04 | 0.02 | 0.10 | 52.56 |
| **EV/TIR contribution (%)** | 8.25 | 0.47 | 60.58 | 1.37 | 1.07 | 16.73 | 11.53 |
| **EVmax contribution (%)** | 1.76 | 39.30 | 7.00 | 2.90 | 22.93 | 25.55 | 0.56 |
| **TIRmax contribution (%)** | 8.95 | 26.23 | 8.78 | 10.14 | 0.04 | 45.15 | 0.71 |
| **EVvel contribution (%)** | 22.50 | 1.99 | 3.28 | 33.72 | 30.42 | 8.08 | 0.01 |
| **EVdur contribution (%)** | 6.89 | 31.88 | 0.94 | 11.79 | 45.50 | 2.99 | 0.01 |

**Supplementary Material C**


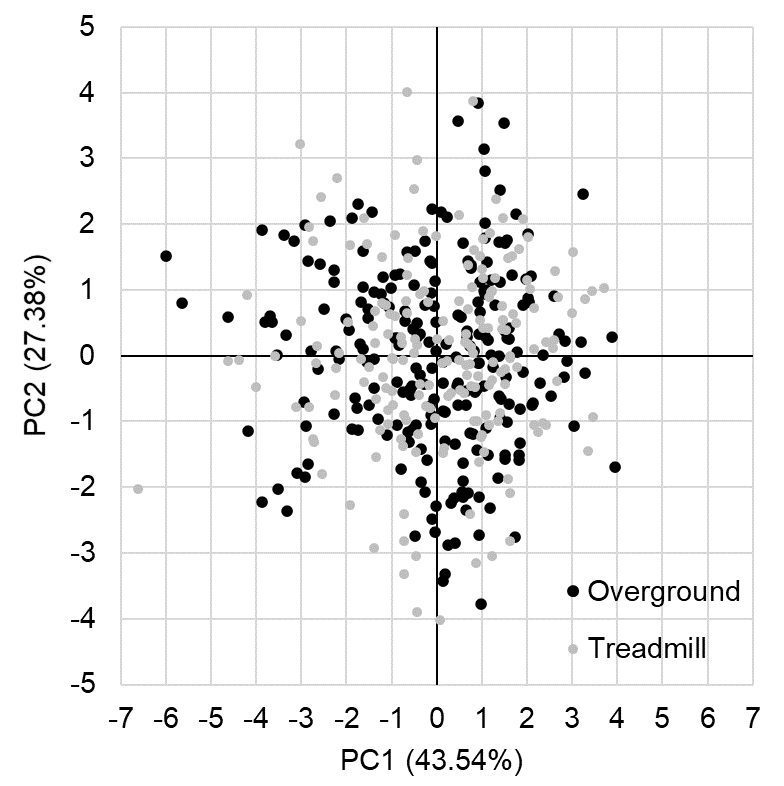


Figure C : Highlighting of the 234 overground and the 190 treadmill runners on the coordinate graph of individuals on the first two PCs.

**Supplementary Material D**


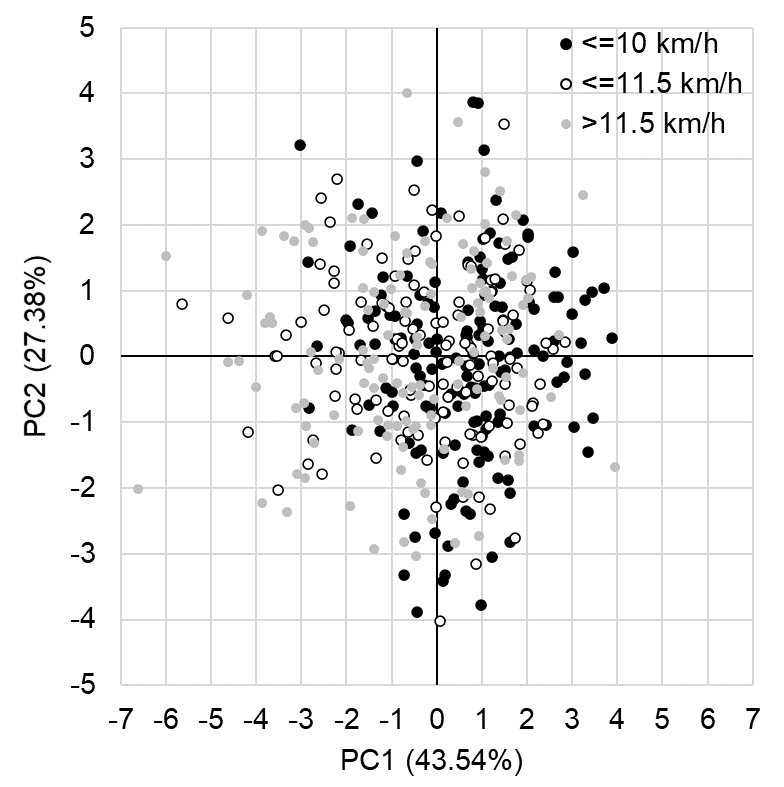


Figure D : Highlighting of the 424 runners with a stratification in three running speeds (<=10 km/h: 162 runners; <=11.5 km/h: 130 runners; >11.5 km/h: 132 runners) on the coordinate graph of individuals on the first two PCs.
